# Supplementary material for: The causal role of circulating inflammatory markers in osteoporosis: a bidirectional Mendelian randomized study
Source: Front Immunol. 2024 Jul 18;15:1412298. doi: 10.3389/fimmu.2024.1412298 (PMC11291241; doi:10.3389/fimmu.2024.1412298)
Supplement: Supplementary file 1 [file DataSheet_1.zip › supplementary material/CIM-OSTEOPOROSIS /GCST90274790.csv_forest.pdf]

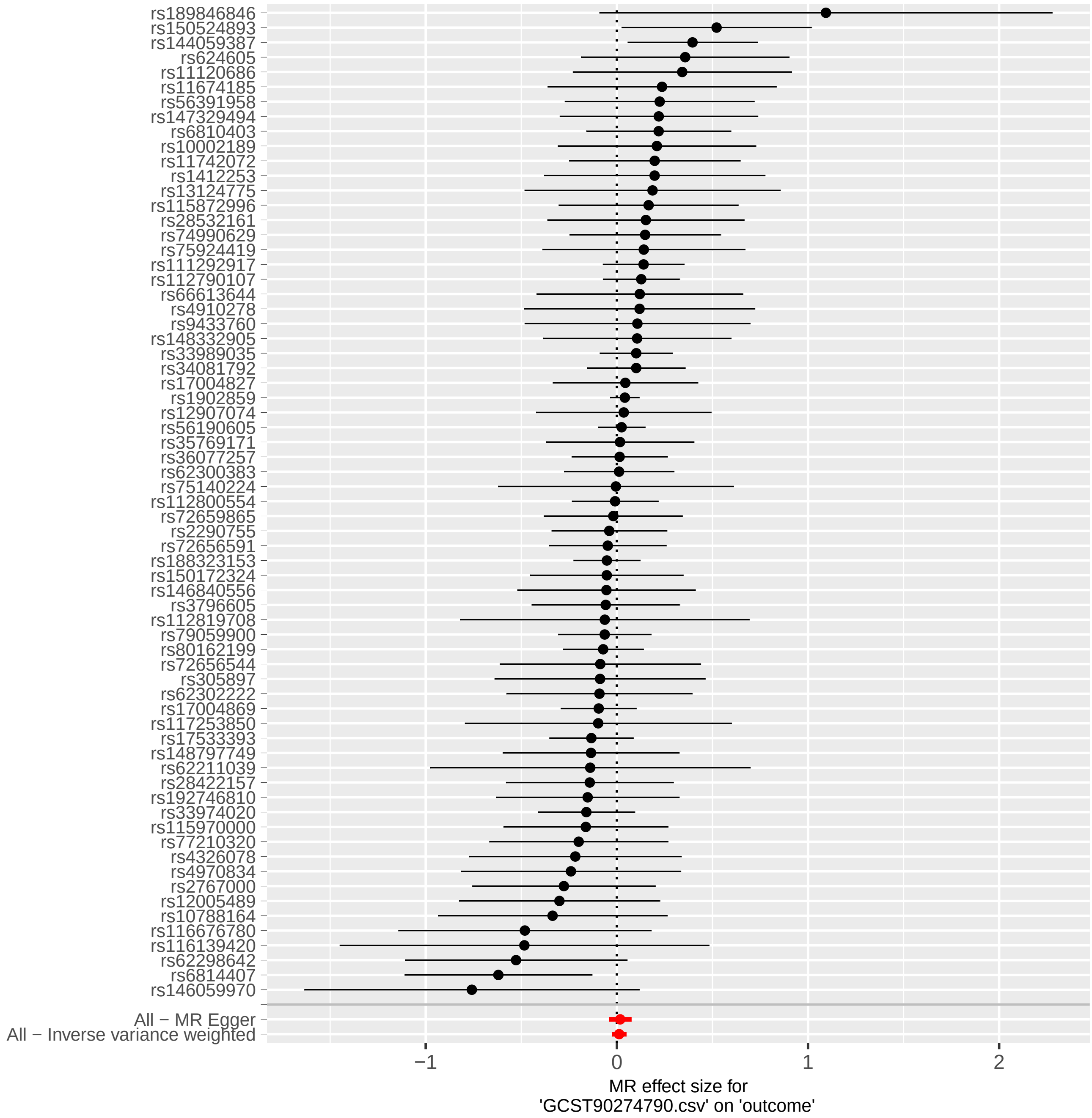
rs189846846
rs150524893
rs144059387
rs624605
rs11120686
rs11674185
rs56391958
rs147329494
rs6810403
rs10002189
rs11742072
rs1412253
rs13124775
rs115872996
rs28532161
rs74990629
rs75924419
rs111292917
rs112790107
rs66613644
rs4910278
rs9433760
rs148332905
rs33989035
rs34081792
rs17004827
rs1902859
rs12907074
rs56190605
rs35769171
rs36077257
rs62300383
rs75140224
rs112800554
rs72659865
rs2290755
rs72656591
rs188323153
rs150172324
rs146840556
rs3796605
rs112819708
rs79059900
rs80162199
rs72656544
rs305897
rs62302222
rs17004869
rs117253850
rs17533393
rs148797749
rs62211039
rs28422157
rs192746810
rs33974020
rs115970000
rs77210320
rs4326078
rs4970834
rs2767000
rs12005489
rs10788164
rs116676780
rs116139420
rs62298642
rs6814407
rs146059970
All − MR Egger
All − Inverse variance weighted
−1
0
1
2
MR effect size for
'GCST90274790.csv' on 'outcome'
